# The complete mitochondrial genome of *Asteroschema tubiferum* (Ophiuroidea: Euryalida: Asteroschematidae)

**DOI:** 10.1080/23802359.2022.2057250

**Published:** 2022-04-01

**Authors:** Zhaoshan Zhong, Minxiao Wang, Haining Wang, Chaolun Li

**Affiliations:** a CAS Key Laboratory of Marine Ecology and Environmental Sciences, and Center of Deep Sea Research, Institute of Oceanology, Chinese Academy of Sciences, Qingdao, China; b Laboratory for Marine Ecology and Environmental Science, Qingdao National Laboratory for Marine Science and Technology, Qingdao, China; c Center for Ocean Mega-Science, Chinese Academy of Sciences, Qingdao, China; d University of Chinese Academy of Sciences, Beijing, China

**Keywords:** *Asteroschema tubiferum*, brittle star, mitogenome

## Abstract

We describe the first mitochondrial genome of a brittle star Asteroschema tubiferum Matsumoto 1911 in family Asteroschematidae. The mitogenome was sequenced and assembled using next-generation sequencing technology, and were 16,361 bp in size with 37 genes containing 13 protein-coding genes, 22 tRNA genes, 2 rRNA genes, and a control region. The phylogenetic tree was constructed based on 13 protein-coding mitochondrial genes of *A. tubiferum* and 26 species in the phylum Echinodermata by RAxML, which showed that it was mostly related to the species in Family Gorgonocephalidae. These results could provide a novel insight to the phylogeny of Ophiuroidea.

Brittle stars, also termed serpent stars are a key group for the study of marine macroecology and biogeography owing to their wide distribution in many marine habitats, especially in the deep waters (Stöhr et al. 2012; O’Hara et al. 2014). *Asteroschema tubiferum* belonging to Euryalida, Asteroschematidae is a common species in the South China Sea and always lives in company with deep-sea corals.

The specimens of *A. tubiferum* were collected by the remotely operated vehicle (ROV) *Faxian* equipped on *RV* Kexue during a cruise in June of 2021 at F site in the South China Sea (22.1163°N, 119.2833°E). The use of animals in this study was approved by the Ethics Committee of the Institute of Oceanology, Chinese Academy of Sciences, and all experiments were conducted in accordance with the guidelines of the committees. The samples were snap-frozen by liquid nitrogen and preserved on board under −80 °C, then transferred to specimen registry of Center of Deep-Sea Research, Institute of Oceanology, CAS (http://www.qdio.cas.cn/, Zhaoshan Zhong, zhongzhaoshan@qdio.ac.cn) under the voucher numbers SCS-CS-2021-09. Total genomic DNA of the brittle star was extracted using the E.Z.N.A. Mollusk DNA Kit (OMEGA Bio-Tek, Norcross, GA, USA). Approximate 10 Gb sequence clean data were generated by Illumina Hiseq (150 bp Pair-end reads, San Diego, USA) in the sequencing center of Novogene. The mitogenome of *A. tubiferum* was de novo assembled using MitoZ pipeline under default parameters except for the clade parameter of echinoderm (Meng et al. 2019) and further annotated on MITOS web server (Bernt et al. 2013) with minor manual revisions based on the ORF prediction results using DNAstar (Swindell and Plasterer 1997). The sequence has been deposited in GenBank under accession number MZ889081. A maximum-likelihood (ML) tree was constructed on the amino acid sequences of concatenated 13 protein-coding mitochondrial genes using the RAxML under PROTGAMMAGTR model (Stamatakis 2014).

The mitogenome of *A. tubiferum* is 16,361 bp in length with a GC content of 32.3%. It is composed of 13 protein-coding genes, 24 transfer RNA genes, and 2 ribosomal RNA genes. A total of 23 mitogenome sequences from class Ophiruoidea and two species from class Asteroidea and one species from class Holothuroidea were downloaded from the GenBank database. The mitogenome sequences of *Acanthaster brevispinus* AB231476, *Archaster typicus* MN052674, and *Holothuria forskali* NC_013884 were used as outgroups. Mitogenome-based phylogenetic relationship among *A. tubiferum* and 23 known mitochondrial genomes of brittle stars (Figure 1) are consistent with the results based on morphological traits. Consistent gene order among taxation suggested a conserved mitochondrial evolution in Class Ophiuroidea. Those results provided background information for further studies on the phylogeny of Ophiuroidea.

**Figure 1.**
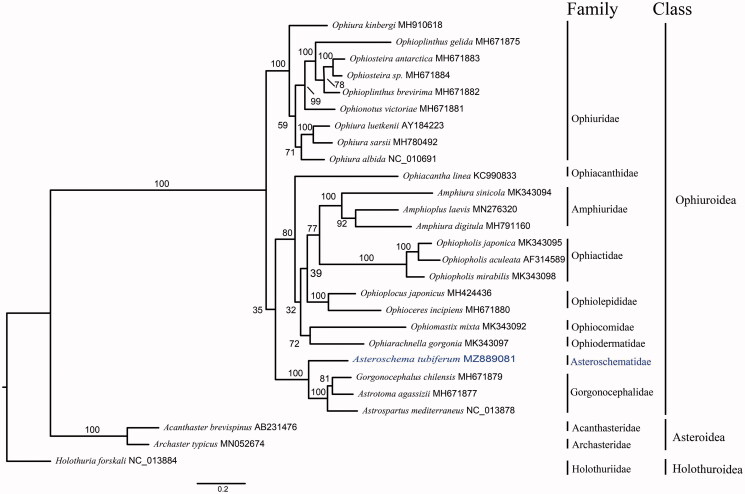
The maximum-likelihood (ML) tree of 24 species of Ophiuroidea class and 3 species in other two classes Asteroidea and Holothuroidea based on the concatenated amino sequences of 13 mitochondrial protein coding genes. The number at each node is the bootstrap support value. The number after species name in GenBank accession number.

## Data Availability

The mitogenome sequence data that support the findings of this study are openly available in GenBank of NCBI (https://www.ncbi.nlm.nih.gov/) under the accession no. MZ889081. The associated BioProject, SRA, and Bio-Sample numbers are PRJNA797927, SRR17641727, and SAMN25041400, respectively.
